# PIDD-dependent activation of caspase-2-mediated mitochondrial injury in E1A-induced cellular sensitivity to macrophage nitric oxide-induced apoptosis

**DOI:** 10.1038/s41420-018-0100-3

**Published:** 2018-09-12

**Authors:** Jay R. Radke, Iris Figueroa, John M. Routes, James L. Cook

**Affiliations:** 1 Division of Infectious Diseases, Department of Medicine, and the Infectious Diseases and Immunology Research Institute, Loyola University Chicago—Stritch School of Medicine, Maywood, IL 60153 USA; 2 Research Section, Edward Hines VA Hospital, Hines, IL 60141 USA; 3 Section of Asthma, Allergy and Clinical Immunology, Department of Pediatrics, Medical College of Wisconsin, Milwaukee, WI 53226 USA; 4 Present Address: Research Section, Boise VA Hospital, Idaho Veterans Research and Education Foundation, Boise, ID 83702 USA

**Keywords:** Cell death and immune response, Cancer

## Abstract

Expression of the adenovirus E1A oncogene sensitizes tumor cells to innate immune rejection by apoptosis induced by macrophage-produced tumor necrosis factor (TNF)-α and nitric oxide (NO). E1A sensitizes cells to TNF-α and NO through two distinct mechanisms, by repressing NF-κB-dependent antiapoptotic responses and enhancing caspase-2 activation and mitochondrial injury, respectively. The mechanisms through which E1A enhances caspase-2 activation in response to NO were unknown. Here, we report that E1A-induced sensitization to NO-induced apoptosis is dependent on expression of PIDD (p53-inducible protein with a death domain) and enhancement of primary immunodeficiency diseases (PIDD) processing for formation of the PIDDosome, the core component of the caspase-2 activation complex. NO-induced apoptosis in E1A-expressing cells did not require expression Bak or Bax, indicating that NO-induced caspase-2-mediated mitochondrial injury does not proceed through the activities of typical, proapoptotic Bcl-2 family members that induce mitochondrial cytochrome C release. These results define a PIDD-dependent pathway, through which E1A enhances casapse-2-mediated mitochondrial injury, resulting in increased sensitivity of mammalian cells to macrophage-induced, NO-mediated apoptosis.

## Introduction

Adenoviruses are nonenveloped, double-stranded DNA viruses that are common respiratory pathogens that generally cause self-limiting infections. In order for adenovirus to replicate in cells, adenoviruses need to usurp the cell cycle machinery. The first adenoviral gene expressed during infection is the early region 1A gene, E1A, which, through its interactions with numerous cellular proteins, modulates both viral and cellular gene expression and is the primary mediator of cell cycle takeover^1^. Through its ability to control cell cycling E1A can immortalize mammalian cells when expressed during abortive infection or stable transfection, like other homologous oncogenes from other DNA tumor viruses and cellular oncogenes^2,3^. Although E1A can immortalize cells, those cells are not tumorigenic in immunocompetent hosts, since expression of E1A sensitizes cells to the apoptosis inducing activities of host immune effector cells, such as natural killer cells, activated macrophages, and cytotoxic T lymphocytes^4^. E1A-induced cellular sensitivity to apoptosis inducing injuries of various types (immunological, chemical, and physical) has been well described^5^. However, the molecular pathways through which this sensitizing activity of E1A is mediated remain to be completely defined^5^.

E1A expression sensitizes cells to the cytolytic mediators of macrophage-mediated apoptosis such as TNF-α, Fas-L, TRAIL, and nitric oxide (NO). Studies have shown that macrophage-induced apoptosis of E1A-expressing cells is mediated primarily by NO^6^. NO inhibits respiration at the level of cytochrome c oxidase (complex IV) in the electron transport chain by competing with oxygen^7,8^. This inhibits generation of the proton motive force and results in decreased cellular adenosine triphosphate (ATP) levels and, if sustained, can result in the loss of mitochondrial membrane potential (MMP). Failure to sustain or recover cellular ATP levels can result in apoptotic cell death^9,10^. We have reported that E1A-negative cells rapidly recover ATP levels following exposure to NO, whereas E1A-positive cells fail to recover ATP, show a loss of MMP and proceed to apoptotic cell death^11^. NO-induced loss of MMP and apoptotic cell death are dependent on caspase activity. Expression of E1A enhances activation of caspase-2 in response to macrophage-NO-induced and chemical-NO-induced apoptosis, which is required for NO-induced apoptosis of E1A-positive cells^11^. However, the mechanisms of this enhancement of NO-induced caspase-2 activation were unclear.

Caspase-2 is the most evolutionarily conserved member of the caspase family, but there are still many questions about its role in the cellular apoptotic pathway^12^. Caspase-2 shares features of both initiator and effector caspases, although it is generally thought to be an initiator caspase. Like other initiator caspases, caspase-2 activation requires dimerization that occurs in high molecular weight complexes. For caspase-2, the predominant activation platform is called the PIDDosome, which is comprised of PIDD, RIP-associated Ich-1/Ced-3 homologous protein with a death domain (RAIDD) and caspase-2^13,14^. Cleavage of PIDD from its full length form to PIDD-C and PIDD-CC is required for PIDDosome-mediated activation of caspase-2^15^. However, the role of PIDD and the PIDDosome in activation of caspase-2 is not absolute^16,17^. Caspase-2 is localized in the nucleus, cytoplasm, ER, golgi, and mitochondria^18–22^. In addition, caspase-2 can induce mitochondrial injury. Caspase-2 can interact with members of the Bcl-2 family that regulate the cellular death response. For example, caspase-2 can cleave Bid, leading to Bid/Bax interaction and resulting in mitochondrial injury^23,24^. Conversely, caspase-2 can mediate mitochondrial injury independently of Bak and Bax^25,26^. Furthermore, caspase-2 can directly injure mitochondria, independently of its enzyme activity and interactions with Bcl-2 family members^27,28^. How caspase-2 is activated and modulates the apoptotic pathways in response to macrophage-NO-induced cell death is unknown.

In this study, we investigated the pathway of caspase-2 activation during apoptosis induced by NO in E1A-expressing cells. Our data show that caspase-2 is the initiator caspase in response to NO injury and is required for NO-induced mitochondrial injury. NO induces processing of PIDD from PIDD-C to PIDD-CC, and both expression and processing of PIDD are required for E1A-induced sensitization to macrophage and chemical-NO-mediated apoptosis and mitochondrial injury. Macrophage-NO-induced and chemical-NO-induced cell death was independent of mitochondrial injury triggered by either Bak or Bax. These results indicate that E1A expression enhances NO-induced caspase-2 activation through the enhancement of PIDDosome activation, resulting in caspase-2 dependent mitochondrial injury through a Bak/Bax independent mechanism.

## Results

### Caspase-2 is the apical mitochondrial injuring caspase in the NO-induced apoptotic pathway

**Fig. 1:**
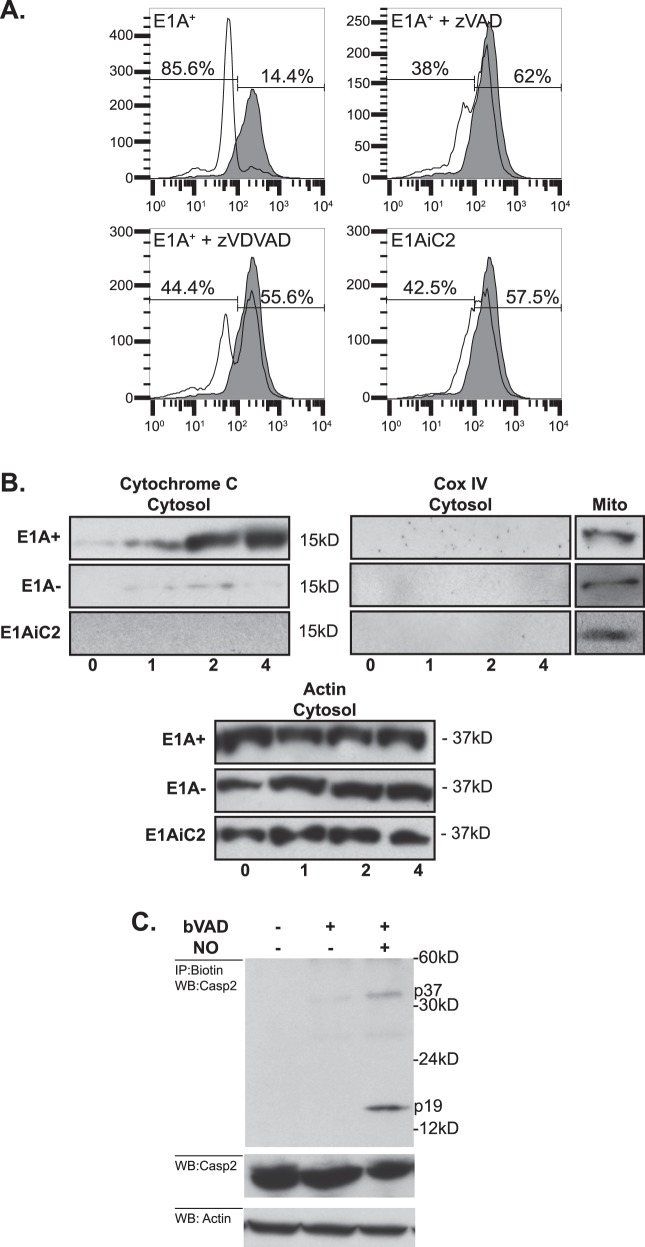
Caspase-2 mediated mitochondrial injury in response to NO. **a** Role of caspase-2 in NO-induced loss of MMP. E1A+ cells were treated with 250 μM DETA-NONOate for 6 h then stained with TMRE to estimate MMP. Loss of MMP by NO-injured (open histogram) compared to uninjured (shaded histogram) E1A-positive cells was dependent on caspase activity (E1A+ zVAD), caspase-2 activity (E1A+ zVDVAD) and expression of caspase-2 (E1AiC2). **b** Mitochondrial release of cytochrome c into the cytosol in E1A-positive (E1A+), E1A-negative, (E1A−) or caspase-2 siRNA-expressing E1A-positive (E1AiC2) cells following treatment with 250 μM DETA-NONOate for the times (h) indicated below the figures. **c** Role of caspase-2 as the initiator caspase in response to NO injury. E1A-positive cells pretreated with or without biotin-VAD (bVAD) and treated with or without 250 μM DETA-NONOate for 4 h

We reported that caspase activity is required for NO-induced loss of MMP^11^. However, the mechanisms through which caspase-2 alters mitochondrial function were unknown. To test the role of caspase-2 in NO-induced mitochondrial injury, we compared the effects of chemical-NO on the MMP of E1A-positive (E1A+) cells, in the absence or presence of either the pan-caspase inhibitor, zVAD, or a caspase-2 inhibitor, zVDVAD. Fig. 1a shows that chemical-NO caused a drop in MMP of E1A-postive cells. Both the pan-caspase inhibitor, zVAD, and the caspase-2-specific inhibitor, zVDVAD, repressed the NO-induced decrease in MMP. To further assess the requirement for caspase-2 in NO-induced mitochondrial injury in E1A-positive cells, we determined the ability of NO to induce apoptosis in caspase-2 knockdown cells (E1AiC2). Similar to E1A+ zVAD cells, NO treatment of E1AiC2 cells resulted in a very small drop in MMP (Fig. 1a). To test whether caspase-2 is a mitochondrial injuring caspase, we examined the release of cytochrome c following NO treatment of E1A+, E1A−, or E1AiC2 cells. Figure 1b, shows that E1A+ cells, when treated with NO, released cytochrome c into the cytosol, whereas E1A− and E1AiC2 cells showed little release of cytochrome c. The retention of Cox IV, an integral mitochondrial membrane protein that remains associated with the mitochondria during apoptosis, in the mitochondrial fraction indicated the purity of the cytosolic fractions. Actin expression served as a loading control. Collectively, these results suggest that, in cells that express E1A, caspase-2 directly injures mitochondria in response to NO.

The role of caspase-2 as an initiator caspase has been controversial. To determine if caspase-2 is the apical caspase activated in response to NO injury, a biotin-zVAD (bVAD) trap pull down assay was performed^29–31^. NO treatment of E1A-expressing cells resulted in the interaction of bVAD with two caspase-2 cleavage fragments p37 and p19 (Fig. 1c). Caspase-8 is the apical caspase activated in response to TNF (ref) and, as expected, TNF treatment did not result in the interaction of bVAD and caspase-2 (data not shown). These data showed that caspase-2 is the apical caspase activated in response to NO injury.

### NO-induced apoptosis requires PIDD expression

**Fig. 2:**
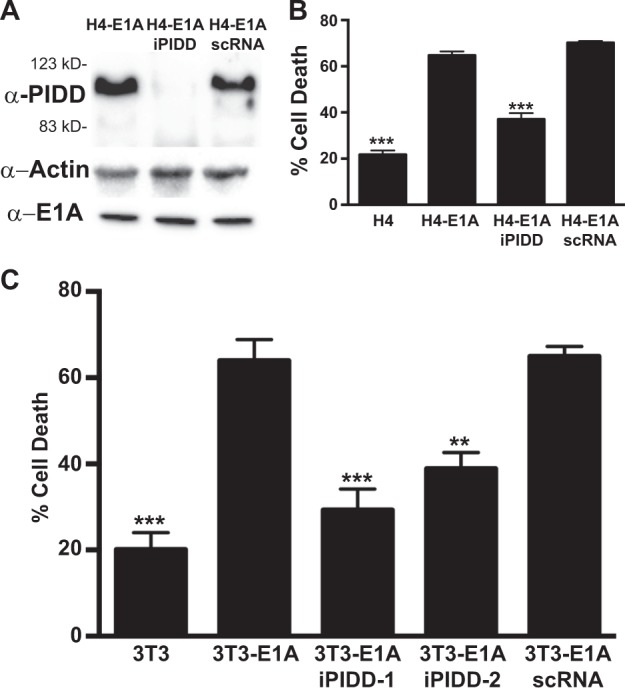
Requirement for PIDD in NO-induced apoptosis. **a** Western blot for the expression of human PIDD, actin and E1A in E1A-positive (H4-E1A), PIDD shRNA E1A+ cells (H4-E1AiPIDD) and E1A+ cells expressing scrambled control shRNA (H4-E1A scRNA). **b** Human fibrosarcoma cells H4, H4-E1A, H4-E1A iPDD, and H4-E1A scRNA treated with 750 μM DETA NONOate for 18 h. Cell viability was determined by MTS staining and expressed as % cell death (mean ± SEM; *n* = 4, ^***^P < 0.001, one-way ANOVA). **c** Mouse NIH-3T3 cells 3T3, 3T3-E1A, 3T3-E1AiPIDD-1 and −2, and 3T3-E1A scRNA treated with 250 μM DETA-NONOate for 18 h. Cell viability was determined by MTS staining and expressed as % cell death (mean ± SEM; *n* = 3, ^***^P < 0.001, ^**^P = 0.0028, one-way ANOVA)

Like other initiator caspases, efficient activation of caspase-2 occurs in a multiprotein activation complex. The predominant activation complex for caspase-2 is the PIDDosome. While the PIDDosome is an efficient activator of caspase-2 and has been shown to be involved in caspase-2 dependent apoptosis, it is not absolutely required for caspase-2 dependent apoptosis. Since we identified caspase-2 as the apical caspase activated in response to NO treatment of E1A-positive cells, we next tested the requirement for PIDD expression in the E1A-mediated sensitization to NO-induced apoptosis. Using shRNA to PIDD, we decreased the expression of PIDD in E1A-positive H4 cells (H4-E1AiPIDD; Fig. 2a). H4-E1AiPIDD cells showed reduced cell death in response to NO treatment compared to H4-E1A cells and H4-E1A-scRNA (a scrambled shRNA control cell line) (Fig. 2b). Similar results were obtained with two different NIH-3T3-E1A-positive, PIDD knockdown cell lines (mouse NIH-3T3-E1AiPIDD) made previously in our laboratory (Fig. 2c)^32^.

### NO-induced processing of PIDD-C to PIDD-CC is required for apoptosis of E1A-positive cells

**Fig. 3:**
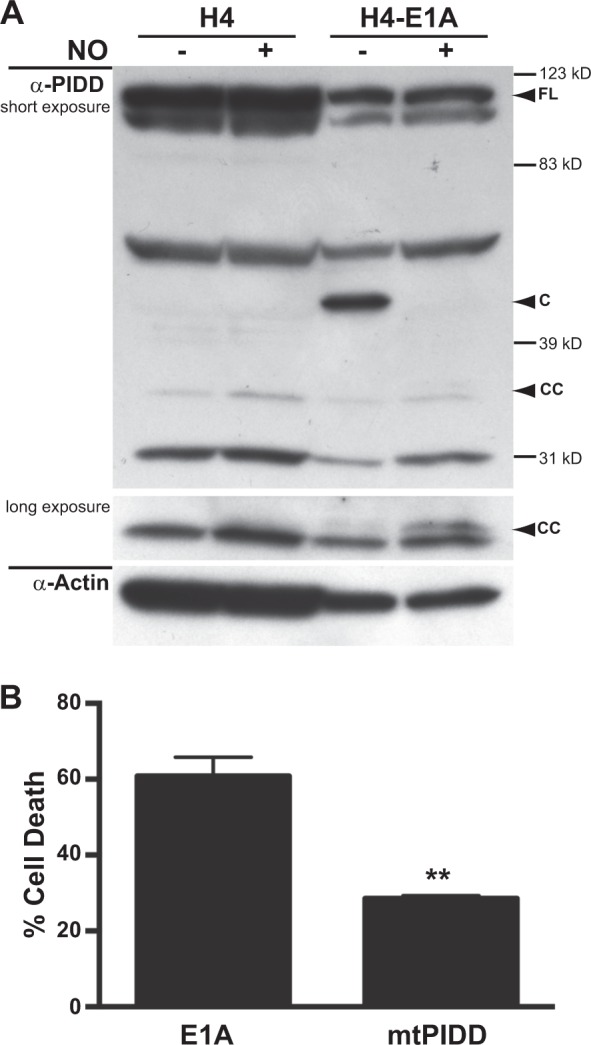
Role of NO-induced PIDD cleavage. **a** Western blot analysis of PIDD and actin in E1A-negative (H4) and E1A-positive (H4-E1A) cells in the absence or presence of DETA-NONOate. **b** Mouse NIN-3T3-E1A+ or E1A+ mtPIDD (S588A) cells were treated with DETA-NONOate for 18 h. Cell viability was determined by MTS staining and expressed as % cell death (mean ± SEM; *n* = 3, ^**^P = 0.003, Student’s two-tailed *t* test)

Formation of the active PIDDosome requires cleavage of PIDD from full length (FL) into two fragments, PIDD-N and PIDD-C. Further processing of PIDD-C to PIDD-CC results in efficient activation of caspase-2^15^. To test whether expression of E1A enhances PIDD processing to PIDD-CC in response to NO, we treated E1A-postive and E1A-negative human fibroblasts (H4 cells) and examined PIDD processing (Fig. 3). Overall, there was no increase in PIDD expression in E1A-positive compared to E1A-negative cells. However, in untreated E1A-postive cells, there was increased spontaneous processing of PIDD-FL to PIDD-C, which was not observed in E1A-negative cells (Fig. 3a). NO treatment resulted in further processing of the PIDD-C to PIDD-CC in E1A-positive, but not E1A-negative, cells. Similar findings were observed in E1A-positive vs. E1A-negative NIH-3T3 cells (data not shown).

Processing of PIDD-C to PIDD-CC occurs at serine 588 (S588). Mutation of S588 to alanine, PIDD-S588A (mtPIDD), results in a PIDD-C product that cannot be processed to PIDD-CC. PIDD-S588A has been shown to both inhibit activation of caspase-2 and prevent caspase-2 mediated apoptosis and, therefore, acts as dominant negative mutation. Expression of mtPIDD in E1A-positive cells repressed NO-induced apoptosis (Fig. 3b). Taken together, these data show that expression and processing of PIDD are required for E1A-mediated sensitization to NO-induced apoptosis.

### PIDD expression and cleavage is required for NO-induced mitochondrial injury

**Fig. 4:**
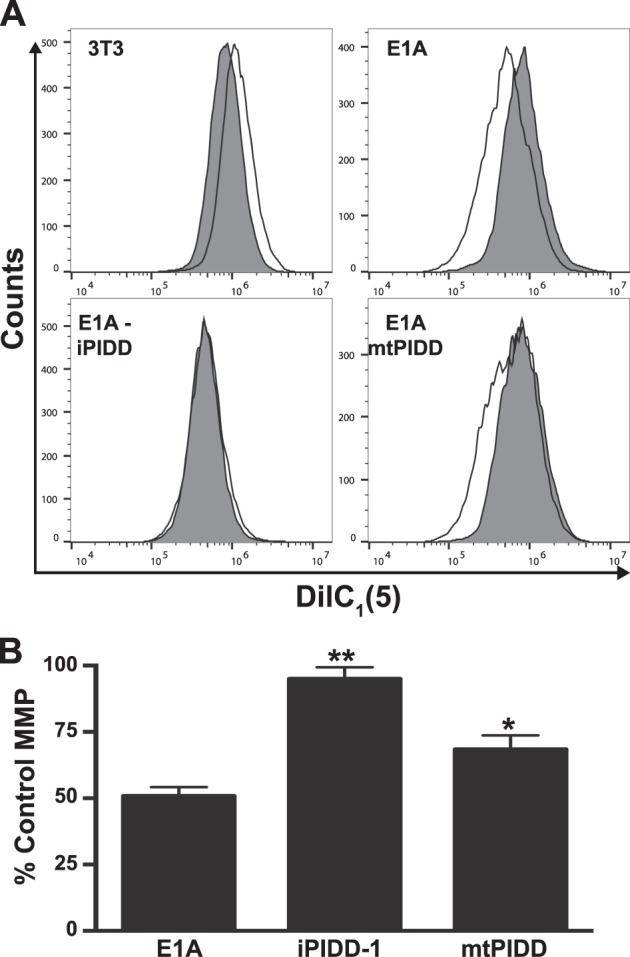
PIDD-mediated mitochondrial injury in response to NO. **a** DilC_1_ (5) staining of NIH-3T3, E1A-positive (E1A), E1A siRNA PIDD (E1A-iPIDD) and E1A PIDD-S588A (E1A-mtPIDD) cells, following treatment with DETA-NONOate for 18 h. Histograms for DETA-NONOate treated cells (unshaded histograms) are overlayed on those for untreated cells (shaded histograms). **b** PIDD-related changes in MMP in response to NO, expressed as a percentage of untreated control cells (mean ± SEM; *n* = 3, ^***^P < 0.001, ^*^P < 0.03, one-way ANOVA)

Since PIDD is required for NO-induced apoptosis, it would be predicted that PIDD would also be required for caspase-2-mediated, NO-induced mitochondrial injury. To test this, we treated both E1AiPIDD and E1AmtPIDD cells with NO and measured mitochondrial membrane potential using DilC staining (Fig. 4). As shown in Fig. 4a, both expression (iPIDD) and cleavage (mtPIDD) of PIDD were required for NO-induced loss of mitochondrial membrane potential. E1AiPIDD cells when treated with NO showed only ~5% loss of mitochondrial membrane potential compared to the 50% loss observed with E1A+ cells (Fig. 4b). E1AmtPIDD cells also showed a significantly reduced decrease in mitochondrial membrane potential compared to E1A+ cells.

### Macrophage-mediated and NO-induced apoptosis of E1A-expressing cells is independent of Bak/Bax expression

**Fig. 5:**
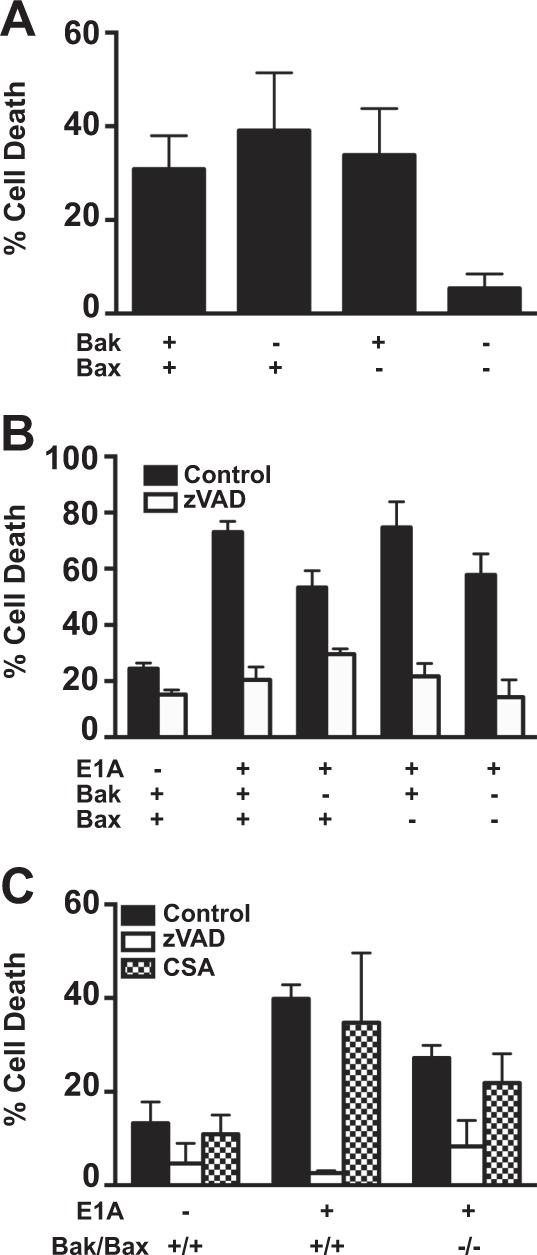
Role of Bak/Bax in macrophage and NO-induced apoptosis. **a** [^3^H]-thymidine-labeled BMK-E1A, BMK-E1A-Bak^−/−^, BMK-E1A-Bax^−/−^, or BMK-E1A-Bak/Bax^−/−^/^−/−^ cells were incubated with ceramide (100 µM) overnight. Supernatants were collected and % specific thymidine release was assessed (mean ± SEM; *n* = 5, ^***^P = 0.001, one-way ANOVA). **b** [^3^H]-thymidine-labeled BMK, BMK-E1A, BMK-E1A-Bak^−/−^, BMK-E1A-Bax^−/−^, or BMK-E1A-Bak/Bax^−/−^/^−/−^ cells were incubated with activated bone marrow-derived macrophages at E:T of 30:1 in the absence or presence of 100 μM zVAD-fmk. After 48 h supernatants were collected and % specific thymidine release was assessed (mean ± SEM; *n* = 3, ^***^P ≤ 0.0001, ^**^P = 0.029, one-way ANOVA). **c** [^3^H]-thymidine-labeled BMK, BMK-E1A, or BMK-E1A-Bak/Bax^−/−^/^−/−^ cells were incubated with DETA-NONOate at 650 µM alone (black bars), with 100 μM zVAD-fmk (white bars) or with cyclosporine A (100 µM) (CSA, hatched bars). After 18 h supernatants were collected and % specific thymidine release was assessed (mean ± SEM; *n* = 3, ^***^P ≤ 0.0005, ^**^P = 0.01, one-way ANOVA)

We have reported that treatment of E1A-expressing cells with NO, results in irreversible mitochondrial injury, as shown by a failure to restore cellular ATP levels following initial NO exposure, as well as a later loss of MMP^11^. Bak and Bax are Bcl-2 family members that can induce mitochondrial injury and cytochrome C release^33^. To determine if Bak/Bax are involved in the caspase-2 dependent macrophage and NO-mediated apoptosis in E1A-expressing cells, we tested E1A-immortalized baby mouse kidney (BMK) cells from either wt (Bak^+/+^/Bax^+/+)^, Bak^−/−^, Bax^−/−^, or Bak^−/−^/Bax^−/−^ mice^34^. As a positive control for Bak/Bax dependent apoptosis we treated wt, Bak^−/−^, Bax^−/−^, or Bak^−/−^/Bax^−/−^ BMKs with ceramide to induce mitochondrial apoptosis. As shown in Fig. 5a, wt BMKs were sensitive to ceramide-induced apoptosis, as were both single knockout Bak^−/−^ and Bax^−/−^ cells, whereas double knockout Bak^−/−^/Bax^−/−^ cells were resistant. To test for Bak/Bax function in macrophage-mediated apoptosis of E1A-positive cells, bone marrow-derived macrophages were isolated from C57Bl/6 mice and activated with lipopolysaccharides (LPS) and IFN-γ and used as killer cells in cytotoxicity assays. Consistent with our previous observations, expression of E1A in wt BMK fibroblasts increased their sensitivity to lysis by activated macrophages, when compared with E1A-negative control BMK cells (Fig. 5b, black bars). Similarly, E1A-positive BMK fibroblasts lacking expression of either Bak, Bax, or both proteins were also significantly more susceptible to cytolysis by activated macrophages than E1A-negative, wt BMK cells. Therefore, E1A-induced cytotoxic sensitivity is independent of expression of either of these proapoptotic, Bcl-2 family members. The apoptotic nature of these macrophage-induced cell death responses was confirmed by the blockade of cytotoxic activity in the presence of the pan-caspase inhibitor, zVAD (Fig. 5b, white bars).

To specifically address whether Bak/Bax are required for E1A-mediated sensitization to NO-induced apoptosis, E1A-positive Bak/Bax-negative BMKs were treated with the chemical-NO donor DETA-NONOate, in the absence or presence of zVAD (Fig. 5c). Consistent with our previous reports, E1A expression enhanced chemical-NO-induced apoptosis of E1A-positive cells (Fig. 5c, black bars), and that E1A-induced NO sensitivity was blocked by zVAD (Fig. 5c, white bars). As had been observed with macrophage-induced apoptosis, Bak/Bax expression was not required for chemical-NO-induced apoptosis (Fig. 5c, Bak/Bax^+/+^ vs. Bak/Bax^−/−^) and required caspase activity, since zVAD inhibited NO-induced apoptosis (Fig. 5c black, control vs. white, zVAD bars).

As shown in Fig. 1a, NO injury results in a loss of mitochondrial membrane potential of E1A-positive cells. Bak/Bax expression is associated with mitochondrial permeability transition pore (MPTP) formation during cellular injuries, resulting in mitochondrial dysfunction and loss of MMP^35–37^. But the results in Fig. 5a–c indicated that Bak/Bax expression is not involved in E1A-induced sensitivity to macrophage or chemical-NO-induced cytotoxicity. These observations suggested the hypothesis that MPTP formation is not required for NO-induced loss of MMP and apoptotic cell death in E1A-positive cells. To test this, NO-induced cytotoxicity of E1A-positive vs. E1A-negative BMKs, either expressing or lacking Bak/Bax, was compared in the presence or absence of cyclosporine A (CsA) that blocks MPTP formation and can, therefore, protect cells against injury-induced cell death^36^. E1A-positive cells were equally sensitive to NO-induced apoptosis, in the presence or absence of CsA, when comparing both Bak/Bax^+/+^ and Bak/Bax^−/−^ cells (Fig. 5c, hatched bars). In addition, activation of casapase-3 following NO treatment of E1A-positive cells was also independent of Bak/Bax expression (not shown). These data collectively indicate that E1A-induced sensitivity to NO-induced, caspase-dependent mitochondrial injury is mediated through a Bak/Bax independent pathway.

## Discussion

The data presented here reveal that the mechanism through which E1A enhances caspase-2-dependent apoptosis of mammalian cells to macrophage-NO-induced cytotoxicity involves increased PIDD processing. These results further define a novel mechanism through which the E1A oncogene sensitizes mammalian cells to innate immune, intrinsic, apoptosis inducing injuries. The data indicate that caspase-2 is the apical caspase required for NO-induced mitochondrial injury. The data are also consistent with our previous reports that E1A alters the cellular metabolic response to NO-induced mitochondrial injury by inhibiting the compensatory glycolytic response to inhibition of cellular respiration, through irreversible damage to mitochondrial membrane integrity^11,38^.

Whether caspase-2 acts as an initiator or effector caspase has been unclear in other systems. Our data show, through multiple lines of evidence, that caspase-2 acts as the initiator caspase activated in response to NO injury of E1A-positive cells. First, both casapse-2 expression and activation are required for NO-induced loss of MMP (Fig. 1a). Second, expression of caspase-2 is required for NO-induced translocation of cytochrome c from the mitochondria to the cytosol (Fig. 1b). Finally, caspase-2 is the apical initiator caspase activated in response to NO-mediated injury, as evidenced by labeling caspase-2 with the irreversible pan-caspase inhibitor, bVAD (Fig. 1c).

The requirements for both the expression and processing of PIDD for E1A-induced cytotoxic sensitivity (Figs. 2 and 3), indicate that the PIDDosome (a high molecular weight complex containing PIDD, caspase-2 and RAIDD) serves as the initiator complex for caspase-2 activation^13^. These data support the conclusion that the cellular mechanism(s) that E1A alters to enhance caspase-2 activation in response to NO injury involves enhancement of PIDD processing. Cleavage of PIDD from FL to PIDD-C is thought to occur through autoproteolysis^15^. How E1A might regulate this activity is unknown. One potential mechanism could involve the increased expression of Hsp90 in E1A-positive cells^39^. Tinel et al.^40^ have shown that Hsp90 binds to PIDD and increases its half life by preventing its degradation by the proteasome. Furthermore, they observed that autoprocessing of PIDD-C to PIDD-CC required the activity of Hsp90^40^. This mechanism would be consistent with the increased autoprocessing of FL PIDD to PIDD-C in E1A-positive cells prior to NO treatment (Fig. 3a, H4-E1A, NO- lane).

It has been postulated that caspase-2 can directly injure mitochondria. Using a cell free model, Roberston and colleagues showed that a VDVAD-sensitive (i.e., caspase-2) activity released from etoposide treated nuclei induces cytochrome c release from purified mitochondria and loss of MMP that is resistant to CsA^41^. Further experiments showed that caspase-2 mediated release of cytochrome c was independent of Bak and Bax^27^. However, direct injury of mitochondria by caspase-2 is not universally observed. Others have shown that caspase-2 cleaves Bid and that t-Bid translocates to the mitochondria to release cytochrome c^23,42–44^. Our data show caspase-2 is required for both cytochrome c release and loss of MMP (Fig. 1a, b). In addition, both macrophage and NO-mediated apoptosis of E1A-expressing cells is independent of the expression of Bak and Bax (Fig. 5b, c). Therefore, NO-induced caspase-2-dependent mitochondrial injury in E1A-positive cells is independent of the activity of these proapoptotic, Bcl-2 family of proteins. These results are consistent with previous observations that expression of the adenoviral protein E1B 19K, which is a viral Bcl-2 homolog and can complement loss of Bcl-2, fails to prevent macrophage-induced apoptosis^45,46^.

We have reported that E1A expression renders cells unable to respond to NO-induced inhibition of cellular respiration and speculated that caspase-2 mediates this blockade of the cellular response to NO injury^11^. Our results showing that caspase-2 is required for the NO-induced loss of MMP (Fig. 1a) and release of cytochrome c into the cytosol (Fig. 1b) indicates that caspase-2, does indeed result in mitochondria injury. We propose that it is this mitochondrial injury that results in the fatal effect of NO on E1A-expressing cells. The inability of CsA to repress NO-induced apoptosis (Fig. 5c) suggests that release of cytochrome c occurs independently of the MPTP in these cells.

We postulate that NO-induced caspase-2 injury of mitochondria results in damage to the mitochondrial membrane that is separate from MPTP opening and that this damage is severe enough that it fails to trigger the compensatory cellular glycolytic response to inhibition of respiration. How caspase-2 might injure mitochondria to such an extent is unclear. It has recently been shown that caspase-2 resides inside mitochondria and is essential for mitochondrial oxidative stress-induced apoptosis^18^. Caspase-2 inside mitochondria is activated in response to injury and can lead to nuclear morphological changes consistent with apoptosis in a cell free model^18^. Similar to our results, it has been reported that other inhibitors of the electron transport chain also require caspase-2 for induction of cell death^18^. It is possible that it is the mitochondrial caspase-2 that results in the irreversible mitochondrial injury and the failure of glycolytic response to restore MMP that is seen in E1A-positive cells following NO exposure. Further studies of mitochondria-associated caspase-2, contrasting the effects of NO injury on E1A-positive and E1A-negative cells, would be required to test this possibility.

## Methods and materials

### Cell lines and cell line characterization

NIH-3T3 cells expressing Ad5 E1A 12S protein (MT12-1) and its derivatives were maintained in DMEM supplemented with antibiotics and 5% calf serum^11,47^. 3T3-E1A+, Caspase-2-shRNA-expressing cells (E1A-iC2) and 3T3-E1A+PIDD-shRNA-expressing cells (E1A-iPIDD-1) have been characterized and were maintained in the presence of G418 and puromycin^11,32^. H4 cells (a subclone of the human fibrosarcoma cell line HT-1080) expressing genomic E1A (H4-E1A and P2AHT2A) and its derivatives were maintained in RPMI 1640 plus antibiotics and 10% fetal bovine serum (FBS)^48^. E1A transformed BMK from wild type, Bak^−/−^, Bax^−/−^, and Bak^−/−^/Bax^−/−^ mice were obtained from Eileen White and were maintained in DMEM with antibiotics and 10% FBS^34^. C-terminal FLAG-tagged PIDD S588A mutant (mtPIDD) was obtained from the laboratory of Jürg Tschopp, cloned into pcDNA3.1 (+)/Hygro (Invitrogen), transfected into E1A+ cells and selected with hygromycin^15,32^. Cell lines were negative for mycoplasma contamination.

### Short hairpin RNA (shRNA) repression of PIDD expression in E1A-positive cells

Human E1A-positive cells were infected with lentiviral particles from Dharmacon (Lafayette, CO) containing shRNAs against human PIDD or a scrambled target sequence. Transduced cells were selected in puromycin, single cell clones were screened and selected for high GFP expression. The selected clones were then tested by western blot for reduced PIDD expression from AbCam (Cambridge, MA) and continued expression of E1A at parental cell levels. The clonal cells used here were generated with the following shRNA target sequence: TCTACTCGCACCTGAAGAA.

### biotin-VAD pull down

The biotin-VAD pull down experiments were done using the method of Tu et al.^29^, with slight modifications. E1A-positive NIH-3T3 cells were labeled with 50 μM biotin-VAD-fmk (Sigma-Aldrich, St. Louis, MO) for 2 h at 37 °C then treated with 250 μM DETA-NONOate for 4 h. After 4 h, cells were lysed in CHAPS lysis buffer, centrifuged at 15,000×*g* for 10 min, and supernatants were boiled for 5 min. Streptavidin-agarose was then added to the supernatants and agitated at 4 °C for 4 h, after which lysates were precipitated and washed three times with CHAPS lysis buffer. Caspase-2 was detected by immunoblotting from both input and immunoprecipitated fractions.

### Isolation of bone marrow-derived macrophages and cytotoxicity assays

Isolation of bone marrow-derived macrophages (BMDM) has been described^6^. Briefly, BMDMs were extracted from femurs and tibias of C57BL/6 mice and were grown in RPMI 1640 with 10% FBS and 20% GM-CSF for 7 days. 24 h before use in macrophage cytotoxicity assays, BMDMs were stimulated with 1 μg/ml LPS (Sigma-Aldrich) and 100 U/ml IFN-γ (R&D Systems, Minneapolis, MN). Macrophage-induced killing was measured by radiolabel release from target cells as described^6,49^. For chemical-NO killing, cells were treated with DETA-NONOate (Cayman Chemical, Ann Arbor, MI), as indicated^11^. As a control for Bak/Bax dependent apoptosis, cells were treated with 100 μM ceramide (Sigma-Alrdich). Release of radiolabel from target cells or MTS assays were used to quantitate cell death^11,32^.

### Analysis of mitochondrial injury

MMP was determined by flow cytometry of cells stained with TMRE or 6 nM MitoProbe DilC_1_ (5) (Invitrogen, Carlsbad, CA) on either a BD LSR or BD Accuri C6 flow cytometer. A non-debris gate was used to remove debris prior to data analysis. Data analysis was performed with FlowJo software.
